# ZFP36 loss-mediated BARX1 stabilization promotes malignant phenotypes by transactivating master oncogenes in NSCLC

**DOI:** 10.1038/s41419-023-06044-z

**Published:** 2023-08-16

**Authors:** Tongjia Zhang, Lizhen Qiu, Jiashun Cao, Qiu Li, Lifan Zhang, Guoshun An, Juhua Ni, Hongti Jia, Shuyan Li, Kailong Li

**Affiliations:** 1 Department of Biochemistry and Biophysics, Beijing Key Laboratory of Protein Posttranslational Modifications and Cell Function, School of Basic Medical Sciences, Peking University Health Science Center, 100191 Beijing, China; 2 Department of Thoracic Surgery, Beijing Tsinghua Changgung Hospital, School of Clinical Medicine, Tsinghua University, 102218 Beijing, China; 3 Department of Research, Beijing Tsinghua Changgung Hospital, School of Clinical Medicine, Tsinghua University, 102218 Beijing, China

**Keywords:** Non-small-cell lung cancer, Oncogenesis

## Abstract

Non-small cell lung cancer (NSCLC) is the most common type of lung cancer, with high morbidity and mortality worldwide. Although the dysregulation of BARX1 expression has been shown to be associated with malignant cancers, including NSCLC, the underlying mechanism remains elusive. In this study, we identified *BARX1* as a common differentially expressed gene in lung squamous cell carcinoma and adenocarcinoma. Importantly, we uncovered a novel mechanism behind the regulation of BARX1, in which ZFP36 interacted with 3’UTR of *BARX1* mRNA to mediate its destabilization. Loss of ZFP36 led to the upregulation of BARX1, which further promoted the proliferation, migration and invasion of NSCLC cells. In addition, the knockdown of BARX1 inhibited tumorigenicity in mouse xenograft. We demonstrated that BARX1 promoted the malignant phenotypes by transactivating a set of master oncogenes involved in the cell cycle, DNA synthesis and metastasis. Overall, our study provides insights into the mechanism of BARX1 actions in NSCLC and aids a better understanding of NSCLC pathogenesis.

## Introduction

Lung cancer is the leading cause of cancer death worldwide. Non-small cell lung cancer (NSCLC) is the most common subtype that accounts for about 85% of all lung cancers, including lung adenocarcinoma (LUAD), lung squamous cell carcinoma (LUSC) and large cell carcinoma (LCLC) [1–3]. Most patients with NSCLC are diagnosed at the advanced stages, often with metastatic disease, resulting in poor prognosis and short overall survival [4]. Thus, a deep understanding of the mechanisms underlying NSCLC tumorigenesis and metastasis is essential to develop more effective diagnostic approaches and therapies for NSCLC.

NSCLC is a highly heterogeneous disease, and the contribution of both tumor cells and immune cells is well recognized. Transcriptome-based subtyping of cancer has identified different subtypes by clustering. However, non-tumor components are usually ignored [5]. ESTIMATE (Estimation of Stromal and Immune cells in Malignant Tumor tissues using Expression data) was developed to not only infer the level of infiltrating stromal and immune cells in tumor tissues but also tumor purity using gene expression data [6]. Accumulating evidence suggests that the ESTIMATE score is closely related to patient survival, metastasis and recurrence, thus serving as a prognostic predictor of cancer [7–10].

The human Barh-like homeobox 1 (*BARX1*) gene at chromosome 9q12 was originally identified to encode a homologous box transcription factor (TF) [11], which plays a key role in the regulation of organ formation and development, such as stomach, intestine, spleen, tooth, and esophagus [12–15]. Most recently, the role of BARX1 in carcinogenesis attracted great attention. One study showed that BARX1 was notably downregulated in human hepatocellular carcinoma (HCC) tissues, which was correlated with poor prognosis [16]. However, increasing evidence has argued that upregulation of the BARX1 is associated with carcinogenesis. For instance, the *BARX1* variant rs11789015 (A>G), which is associated with decreased expression of BARX1 mRNA and protein, confers a decreased risk of esophageal squamous cell carcinoma (ESCC) [17]. It also revealed that highly expressed BARX1 can act as a carcinogen to promote cell viability, invasion, and migration in endometrial carcinoma (EC) partly through the regulation of the ERK/MEK pathway [18]. Likewise, bioinformatics algorithm and differentially expressed TFs’ analysis show that BARX1, as well as DLX4, play an oncogenic role in clear cell renal cell carcinoma (ccRCC) by promoting proliferation and epithelial–mesenchymal transition [19]. Genome-wide identification of TFs critical to lung carcinogenesis has revealed that 10 potential oncogenic TFs were required for NSCLC, including BARX1 [20]. Altogether, it suggests that dysregulation of BARX1 may contribute to carcinogenesis. However, little is known about the mechanism behind it.

RNA-binding proteins (RBPs) play a central role in post-transcription by modulating mRNA metabolism and function. Dysfunction of RBPs affects a diverse range of physiological and pathological processes, including cancer. The zinc-finger protein 36 (ZFP36), also known as tristetraprolin (TTP), Nup475, G0S24 and TIS11, is the prototypic member of the TIS11/ZFP36 family of RBPs, composed of ZFP36, ZFP36L1 and ZFP36L2. These family members have been characterized by highly conserved tandem CCCH zinc-finger RNA-binding domains [21]. ZFP36 acts in the regulation of targeted gene expression at the transcriptional level, post-transcriptional level and translational levels, in which the promotion of AU-rich mRNA decay by ZFP36 at the post-transcriptional level is the most common and classical regulation mechanism [22]. The AU-rich mRNAs are a class of mRNAs bearing AU-rich elements (ARE) in their 3ʹ untranslated regions (3ʹUTR). So-called ARE is AUUUA pentamer in AU context, e.g., UAUUUAU or UUAUUUAU or overlapping repeats of the pentamer [23]. The ARE-mRNAs have been estimated to be approximately 10–15% of all transcripts, involved in a variety of physiological and pathological processes such as cellular proliferation, development, and many diseases, including chronic inflammatory diseases and cancer [22, 23]. ZFP36 promotes ARE-mRNA decay, thereby reducing gene expression by binding to the AUUUA, UAUUUAUU octamer, UUAUUUAUU nonamer or other AU/U-rich sequences in targeted mRNAs. It seems that the association of the UAUU semi-binding site with ZFP36-induced change of mRNA abundance is stronger than that of AUUUA pentamers [24]. ZFP36 has previously been described as a tumor suppressor whose expression was downregulated or lost in human cancer cell lines [25] and various types of cancer such as prostate cancer [26, 27], colorectal cancer [28], breast cancer, pancreatic cancer, hepatocellular carcinoma (HCC), malignant melanoma, and malignant glioma [22, 23]. However, the suppressive role of ZFP36 in NSCLC is unclear, and the functional link between ZFP36 and BARX1 is unknown.

In this study, we investigated the functional interplay between ZFP36 and BARX1 and the biological function of ZFP36-targeted BARX1 in NSCLC. We demonstrated that BARX1 was upregulated and functioned as an oncogene, whereas ZFP36 was downregulated and acted as a suppressor in NSCLC cell lines and clinical tumor tissues. We deciphered a novel mechanism for the regulation of BARX1, in which ZFP36 mediated *BARX1* mRNA destabilization by binding to the 3’UTR of *BARX1* mRNA. Moreover, downregulation of ZFP36 led to aberrant high expression of BARX1, followed by activation of downstream master oncogenes CDC20, CDC45, TRIM37 and MMP-9, thereby promoting in vitro proliferation, migration, invasion and in vivo tumorigenicity of NSCLC cells.

## Results

### *BARX1* is a common DEG in NSCLC

**Fig. 1:**
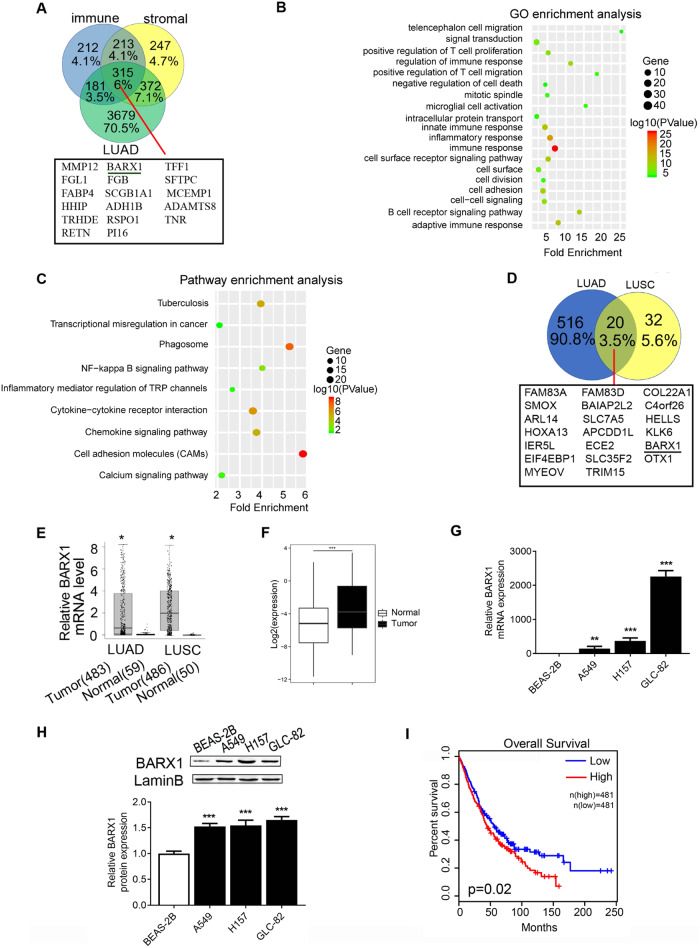
Identification of BARX1 as a commonly upregulated differential expressed gene in lung cancer. **A** Screening of common DEGs based on LUAD RNA-seq data from the TCGA database and ESTIMATE database. **B** GO function enrichment analysis. **C** Enrichment analysis of KEGG pathway. **D** Venn diagram showing 20 DEGs commonly in LUAD and LUSC, the DEG screening and its diagnostic and prognostic value analysis were carried out using the RNA-seq data of LUAD and LUSC in the TCGA database combined with clinical data. **E** The expression level of *BARX1* mRNA in LUAD (483 cases) and LUSC (486 cases) tissues vs normal tissues based on the RNA-seq data from the TCGA database. **F** *BARX1* mRNA level in NSCLC tissues compared to the matched normal adjacent tissues of lung cancer patients (*n* = 23). **G** The expression level of *BARX1* mRNA in normal lung cells BEAS-2B (human bronchial epithelial cell line) and human NSCLC cell lines A549, H157 and GLC-82 (*n* = 3). **H** The expression level of BARX1 protein in normal lung cells and NSCLC cells (*n* = 3). **I** Overall survival analysis showing that high BARX1 expression is associated with a low survival rate. Data were collected from the TCGA (The Cancer Genome Atlas) database, with 962 NSCLC cases which were divided into high BARX1 group (*n* = 481) and low BARX1 group (*n* = 481) and analyzed using the GEPIA online tool.

To identify the key regulators involved in NSCLC progression by modulating tumor cells and/or tumor-associated immune cells, we first downloaded LUAD and LUSC RNA-seq datasets of 483 LUAD cases and 486 LUSC cases from The Cancer Genome Atlas (TCGA) database, and combined with ESTIMATE algorithm derived immune and stromal scores to identify 315 DEGs in LUAD (Fig. 1A, Supplementary File 1). GO (gene ontology) and KEGG (Kyoto Encyclopedia of Genes and Genomes) pathway enrichment analyses revealed that these DEGs were mainly related to signal pathways in cancers, inflammation, cell adhesion and immune response (Fig. 1B, C). Next, based on LUAD and LUSC RNA-seq data from the TCGA database along with prognostic analysis, 20 DEGs commonly expressed in both LUAD and LUSC with diagnostic and prognostic values were obtained (Fig. 1D), among which three transcription factors were included (HOXA13, BARX1 and OTX1). BARX1 stood out because of the bigger differences between the LUAD and LUSC in terms of gene expression. Thus, we decided to continuously investigate the role and the regulatory mechanism of BARX1 in NSCLC.

### BARX1 is highly expressed in NSCLC patient samples

To further investigate the role of BARX1 in NSCLC, we first analyzed the expression level of BARX1 in tumor tissues from 486 LUSC patients and 483 LUAD patients using GEPIA (http://gepia.cancer-pku.cn/). We found that the mRNA level of *BARX1* was significantly upregulated in LUAD and LUSC tissues compared to normal tissues, respectively (Fig. 1E). No difference in BARX1 expression was found between different stages of the LUAD and LUSC tissues (Supplementary Fig. S1). Further, we examined the expression levels of *BARX1* mRNA and protein in lung cancer tissues from 23 patients with LUAD, LUSC and lung cancer cell lines by RT-qPCR or Western blotting. The results showed that the relative expression level of *BARX1* mRNA in lung cancer tissues increased by about tenfold compared to matched normal adjacent tissues (Fig. 1F). Likewise, the relative expression levels of *BARX1* mRNA in A549, H157 and GLC-82 lung cancer cells were significantly increased compared to noncancer BEAS-2B cell (Fig. 1G). Consistently, Western blotting showed that the expression of BARX1 protein in A549, H157 and GLC-82 was increased compared to BEAS-2B (Fig. 1H). Taken together with the results of database analyses (Fig. 1A–E) and our expression assays of lung cancer tissues from patients, we conclude that BARX1 is upregulated in NSCLC tissues and NSCLC cell lines. To ascertain the clinical significance of BARX1 expression, we performed Kaplan–Meier survival analysis using GEPIA, and it showed that the high expression of BARX1 had a poor survival rate in prognosis analysis (Fig. 1I), indicating that BARX1 acts as an oncogene in NSCLC.

### BARX1 is negatively post-transcriptionally regulated by RBP ZFP36

**Fig. 2:**
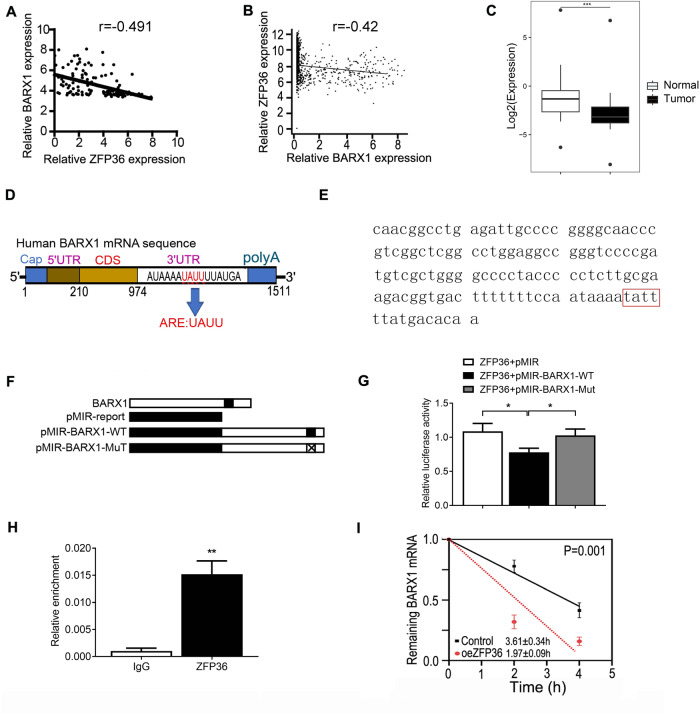
ZFP36 regulates BARX1 by binding to the AU enrichment elements in *BARX1* 3’UTR. **A** The correlation between ZFP36 and BARX1 expression in different lung cancer cells (*n* = 123) from the CCLE database (r = −0.446, *p* < 0.001). **B** The correlation between ZFP36 and BARX1 expression in 969 lung cancer samples and 685 normal samples from TCGA and GTEx database (r = −0.42, *p* < 0.001). **C** The relative expression of *ZFP36* mRNA in cancer tissues and the matched normal adjacent tissues of lung cancer patients (*n* = 14). **D**, **E** Structure pattern diagram of BARX1. AU enrichment elements at position 1497–1550 in the 3’UTR of BARX1, which are the binding sites of ZFP36. **F** Construction of wild-type and mutant dual-luciferase reporter vectors for ZFP36 binding sites. **G** Dual-luciferase reporter gene assay showed that the relative luciferase activity of the wild-type plasmid is reduced but not of the mutant plasmid (*n* = 3). **H** RIP experiment shows that the ZFP36 antibody can pull down more *BARX1* mRNA compared with IgG antibody (*n* = 3). **I** The half-life of *BARX1* mRNA was evaluated in A549 cells when transfected with pcDNA3-ZFP36 plasmid. Data are the means ± SD from three independent experiments. *p < 0.05, **p < 0.01, ***p < 0.001.

BARX1 expression was shown to be precisely regulated in space and time [29, 30]. RNA-binding proteins (RBPs) are critical effectors of gene expression, and as such, their malfunction underlies the origin of many diseases, including cancer [31]. We postulated that the dysregulation might occur through specific RBPs that target *BARX1* mRNA. To test the hypothesis, we explored the candidate RBPs using an online tool Starbase [32], and the investigation resulted in 13 candidate RBPs that may target *BARX1* mRNA. Further analysis showed that four candidate RBPs showed significant dysregulation in TCGA datasets (IGF2BP3, ZFP36, CBX7, KHDRBS2); more importantly, only ZFP36 expression correlated with BARX1 expression in both CCLE (Fig. 2A) and TCGA (Fig. 2B) database in NSCLC by Spearman’s correlation analysis. We then decided to test the possibility that ZFP36 regulates BARX1 through binding to *BARX1* mRNA.

Since ZFP36 expression negatively correlated with BARX1 expression, we then investigate whether ZFP expression is downregulated in NSCLC. We performed RT-qPCR, and the results showed significant downregulation of *ZFP36* mRNA in NSCLC tissues of patients compared to matched normal adjacent tissues (Fig. 2C). As ZFP36 is capable of binding and targeting ARE-mRNAs for rapid degradation, we next searched the AU/U-rich sequence and found a UAUU semi-binding site [24] in the 3’UTR (1497–1500) of *BARX1* mRNA (Fig. 2D, E), which might be recognized by the RBP ZFP36.

To determine if ZFP36 interacts directly with *BARX1* mRNA by targeting the UAUU semi-binding site, dual-luciferase reporter assays were performed using the pMIR-Report-BARX1-WT and pMIR-Report-BARX1-MT plasmids, in which a 537-bp fragment of a human BARX1 DNA sequence containing wild-type or mutated binding site for ZFP36 was inserted into downstream of the *Luc* gene (Fig. 2F). A549 cells were then co-transfected by the wild-type or mutated reporter plasmid with the pcDNA3-ZFP36 expression plasmid. Forty-eight hours after transfection, the luciferase activity was assayed, and it showed that ZFP36 overexpression significantly inhibited the luciferase activity in pMIR-Report-BARX1-WT transfected A549 cells, but not pMIR-Report-BARX1-MT transfected A549 (Fig. 2G). The inhibition of reporter enzyme activity should be attributed to the binding of ZFP36 to the 3′UTR of *BARX1* mRNA. To further validate that ZFP36 interacted with the 3′UTR of *BARX1* mRNA, RNA immunoprecipitation (RIP) assay was performed, and it showed that ZFP36 antibody could pull down more *BARX1* mRNA, while IgG could not do so (Fig. 2H). Taken together, it suggests that ZFP36 interacts with *BARX1* mRNA by preferentially binding to the UAUU semi-binding site in 3’UTR of *BARX1* mRNA and inhibits its expression.

ZFP36 suppresses targeted gene expression by promoting ARE-mRNA degradation or decay [22, 23]. To further demonstrate the regulation of *BARX1* mRNA by ZFP36, we tested the half-life of *BARX1* mRNA in A549 cells transfected with pcDNA3-ZFP36 expression vector. We observed that overexpression of ZFP36 cut short the half-life of *BARX1* mRNA from 3.6 h to about 1.9 h (Fig. 2I). To further confirm the specific interaction of ZFP36 with 3’-UTR sequence of BARX1, we performed RNA-Protein pull-down assay. The results showed that the wild-type probe with UAUU half-site specifically enriched ZFP36 but not the mutant probe (Supplementary Fig. S2). These results indicate that ZFP36 regulates the expression of BARX1 by destabilizing *BARX1* mRNA.

### ZFP36 negatively regulates BARX1 in NSCLC cells

**Fig. 3:**
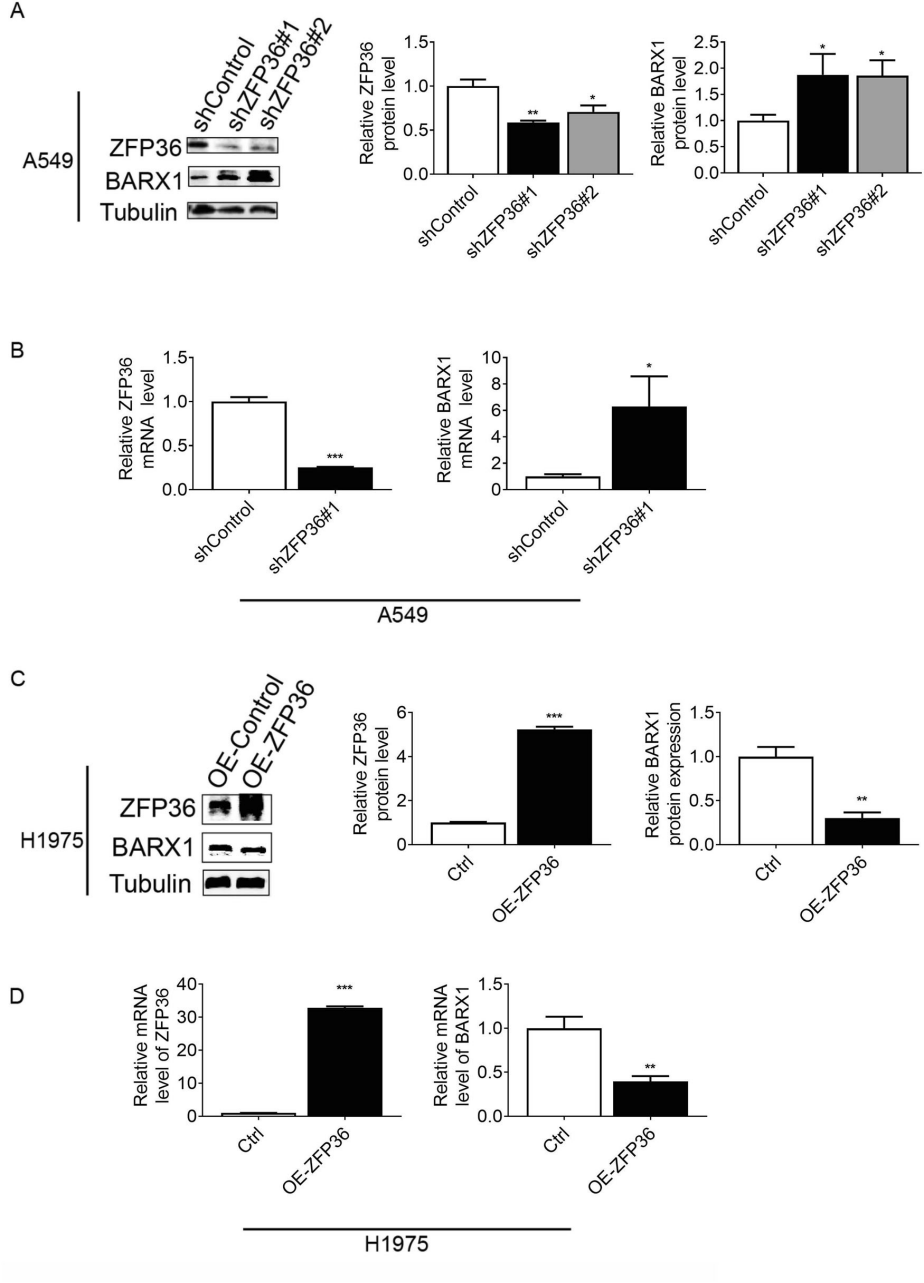
Overexpression or knockdown of ZFP36 negatively regulates BARX1. **A** Western blot results verified the stable knockdown of ZFP36 and upregulation of BARX1 in A549 cells. **B** Upregulation of *BARX1* mRNA induced by the stable knockdown of ZFP36 in A549 cells was verified by RT-qPCR. **C** Western blot results showed that overexpression of ZFP36 reduces the BARX1 protein level in H1975 cells. **D** Overexpression of ZFP36 reduces the *BARX1* mRNA level in H1975 cells, verified by RT-qPCR. Data are the means ± SD from three independent experiments. *p < 0.05, **p < 0.01, ***p < 0.001.

To further define the regulation of BARX1 by ZFP36 in NSCLC, we constructed two stable cell lines expressing shRNA targeting ZFP36. It showed that the levels of BARX1 protein were significantly increased compared to the control (Fig. 3A, right panel) when silencing ZFP36 by shRNAs (Fig. 3A, middle panel). RT-qPCR results revealed that the knockdown of *ZFP36* in A549 increased the level of BARX1 mRNA by approximately 30% compared with control (Fig. 3B), which was also demonstrated by RNA interference (RNAi) experiments (Supplementary Fig. S3). We then further investigated the generality of ZFP36-mediated negative regulation of BARX1, and it showed that the protein and mRNA levels of BARX1 were reduced by about 68% and 50%, respectively, in ZFP36-overexpressed H1975 cells (Fig. 3C, D). These results indicate that BARX1 is negatively regulated by ZFP36 in NSCLC cells.

### Knockdown of BARX1 inhibits proliferation, migration and invasion of NSCLC cells

**Fig. 4:**
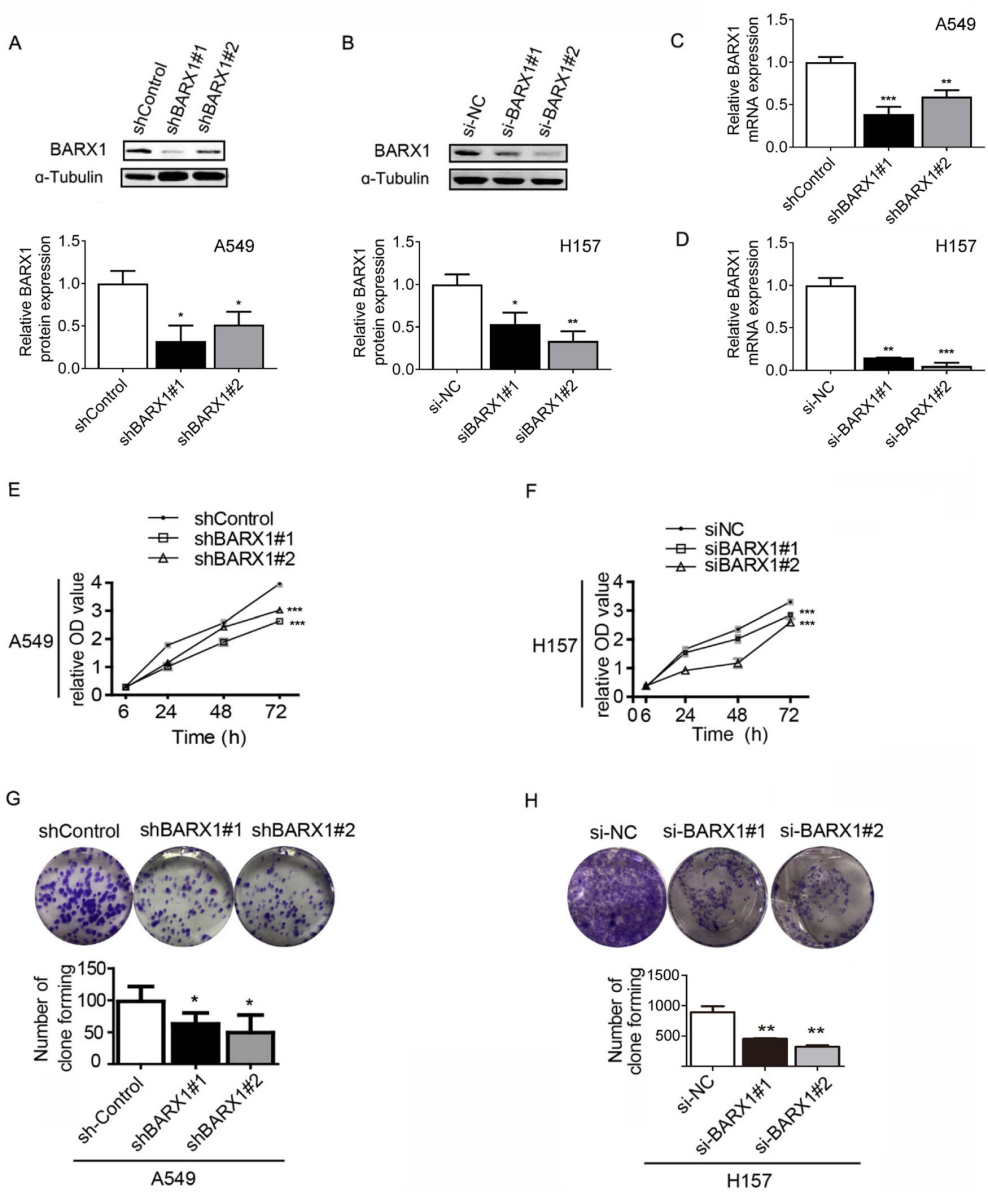
Knockdown of BARX1 inhibits the proliferation of lung adenocarcinoma cells. Knockdown of BARX1 by shRNA in A549 cells was verified by Western Blot (**A**) and RT-qPCR (**C**). Knockdown of BARX1 by siRNA in H157 cells was verified by Western blot (**B**) and RT-qPCR (**D**). **E** Knockdown of BARX1 significantly reduced the cell proliferation rate in A549. The cell proliferation was detected by CCK8 assay at 0, 24, 48 and 72 h. **F** Knockdown of BARX1 in H157 cells reduced the cell proliferation rate assayed by CCK8 assay. **G** Knockdown of BARX1 resulted in decreased anchorage-independent growth capacity in A549 with colony formation assay. **H** Knockdown of BARX1 resulted in decreased anchorage-independent growth capacity in H157 with colony formation assay. Data are the means ± SD from three independent experiments. *p < 0.05, **p < 0.01, ***p < 0.001.

To investigate the role of BARX1 in NSCLC, we constructed two stable cell lines expressing shRNA targeting *BARX1*. The stable knockdown efficiency of the BARX1-shRNA#1 (shBARX1#1) and BARX1-shRNA#2 (shBARX1#2) was verified by Western blotting and RT-qPCR (Fig. 4A, C). We also transfected siRNAs against *BARX1* (siBARX1#1 and siBARX1#2) into LUAD H157 cells and verified the knockdown efficiency by Western blotting and RT-qPCR (Fig. 4B, D). Then we investigated the effects of *BARX1* knockdown on cancer cell growth and proliferation using these cells. CCK8 assays showed that stable knockdown of *BARX1* by shBARX1 in A549 cell and transient *BARX1* knockdown by siBARX1 in H175 cells significantly decreased the survival rate of the cells by about 34% and 39% (of control) at 72 h, respectively (Fig. 4E, F). Colony formation assays showed that knockdown of BARX1 by shBARX1#1 or shBARX1#2 led to a 36% or 50% decrease of colony formation number in A549, compared with the descrambled shRNA transfected A549 (shControl cells) (Fig. 4G). Likewise, in H157 cells, the number of clones was reduced by about 36% or 53% by siBARX1#1 or siBARX1#2 (Fig. 4H).

**Fig. 5:**
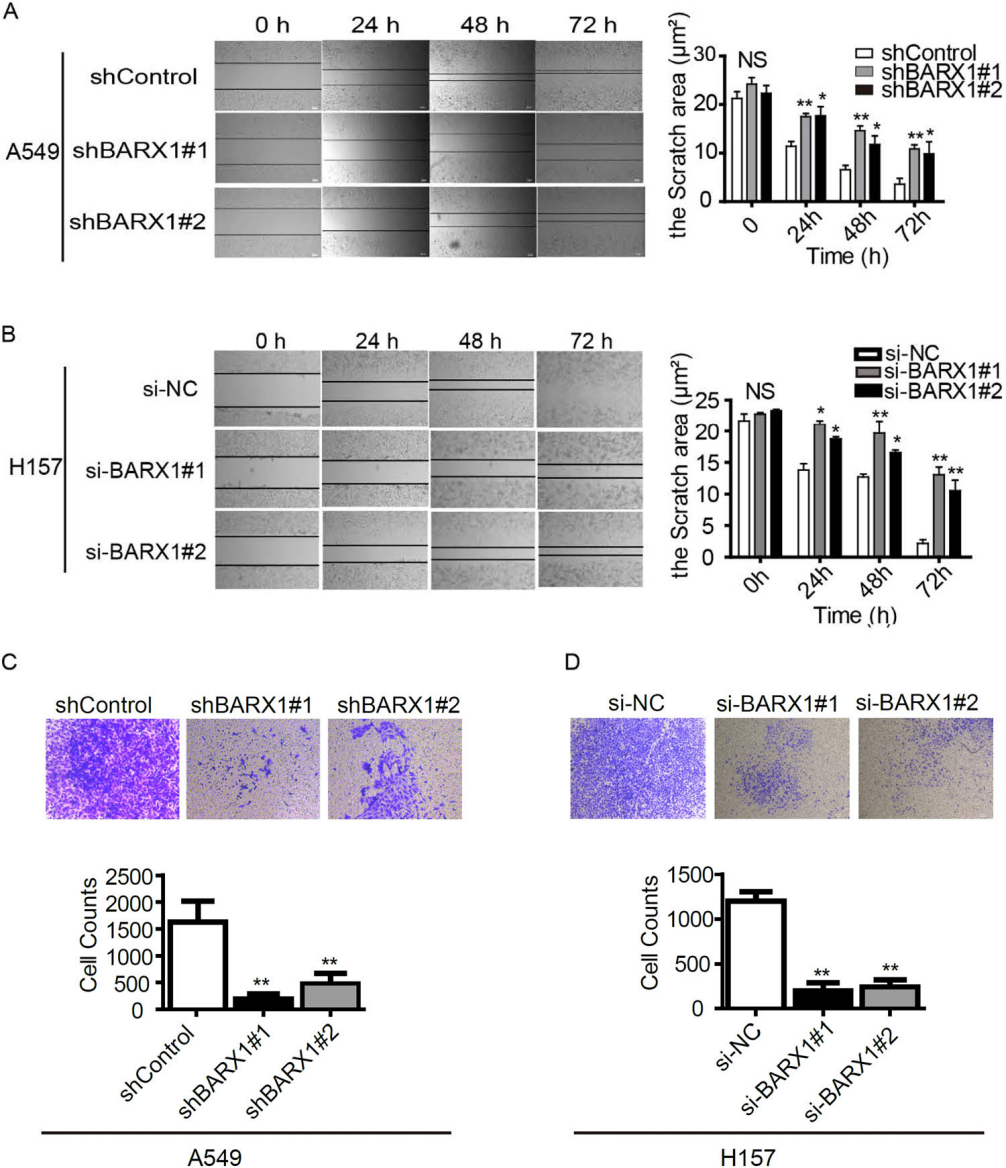
Knockdown of BARX1 inhibits the migration and invasion of lung cancer cells. **A** Knockdown of BARX1 significantly decreased the healing rate of the scratch area in A549 cells. **B** Knockdown of BARX1 significantly decreased the healing rate of the scratch area in H157 cells. **C** Knockdown of BARX1 significantly decreased the cell invasion ability in A549 cells detected by Transwell assay. **D** Knockdown of BARX1 significantly decreased the cell invasion ability in H157 cells detected by Transwell assay. Data are the means ± SD from three independent experiments. *p < 0.05, **p < 0.01, ***p < 0.001.

To further explore whether BARX1 affected migration and invasion of lung cancer cells, wound-scratch tests and transwell experiments were performed. The wound-scratch results showed that knockdown of *BARX1* by shBARX1 or siBARX1 markedly decreased the healing rate of the scratch area compared with the control in A549 (Fig. 5A) and H157 (Fig. 5B) cells. Transwell matrigel invasion assays showed that knockdown of *BARX1* by shRNAs in A549 or by siRNAs in H157 notably suppressed cell invasion compared with the control (Fig. 5C, D). These results indicate that the knockdown of *BARX1* inhibits, but the expression of *BARX1* promotes NSCLC cell proliferation, migration and invasion.

### Knockdown of BARX1 suppresses downstream master oncogenes

**Fig. 6:**
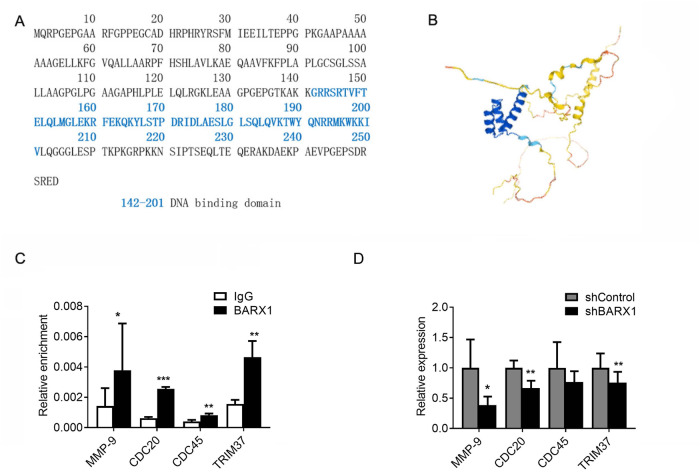
Knockdown of BARX1 suppresses its downstream genes’ expression. **A** DNA binding domain of BARX1 was analyzed by the JASPAR database. **B** AlphaFold structure prediction showed the structure of BARX1 with a high per-residue confidence score (>90) of 142–201 DNA binding domain (three blue helix). **C** ChIP-qPCR experiments showed that MMP-9, CDC20, CDC45 and TRIM37 are transcriptionally regulated by BARX1. **D** Knockdown of BARX1 leads to the reduction of mRNA levels of MMP-9, CDC20, TRIM37, and CDC45 in H157 and A549 cells. Data are the means ± SD from three independent experiments. *p < 0.05, **p < 0.01, ***p < 0.001.

Why BARX1 expression promotes, but BARX1 knockdown inhibits cell proliferation, migration and invasion of lung cancer cells? To answer this question, we speculated that BARX1 as a transcription factor might transactivate its downstream genes involved in these phenotypes. Through JASPAR database (http://jaspar.genereg.net/), the DNA binding site of BARX1 was analyzed (Fig. 6A), and the structure of BARX1 was shown based on AlphaFold structure prediction, of which the 142–201 DNA binding domain has a high per-residue confidence score (>90) (Fig. 6B). Next, we performed in silico prediction for the target genes of BARX1 (Supplementary File 2), among 86 target genes we identified, four master oncogenes have been shown to be drivers in tumor progression, including *CDC20* (cell division cycle 20), *CDC45* (cell division cycle 45), *TRIM37* (tripartite motif containing 37) and *MMP-9* (matrix metalloproteinase-9) which are involved in cell proliferation, migration and invasion, respectively.

To demonstrate if these genes were the target genes of BARX1, chromosome immunoprecipitation (ChIP) assays were performed. A549 cell lysates were immunoprecipitated with anti-BARX1 antibody or IgG (control), and the genomic DNA fragments containing the regions of these four promoters in the precipitates were amplified by RT-qPCR, respectively. The results from the ChIP assays showed that the TF BARX1 were recruited to the promoters of the *CDC20*, *CDC45*, *TRIM37* and *MMP-9* genes (Fig. 6C), indicating BARX1 binding to these promoters. To demonstrate if the BARX1 binding could activate these genes, the mRNA levels of *CDC20*, *CDC45*, *TRIM37* and *MMP-9* in *BARX1* knockdown-A549 and -H157 cells were determined by RT-qPCR. The results showed that the knockdown of *BARX1* significantly decreased mRNA levels of *CDC20*, *CDC45*, *TRIM37* and *MMP-9* genes (Fig. 6D). These data indicate that BARX1 promotes cellular proliferation, migration and invasion of lung cancer cells by activating the downstream master oncogenes including *CDC20*, *CDC45*, *TRIM37* and *MMP-*9 genes.

### Knockdown of BARX1 inhibits tumorigenicity in mouse xenografts

**Fig. 7:**
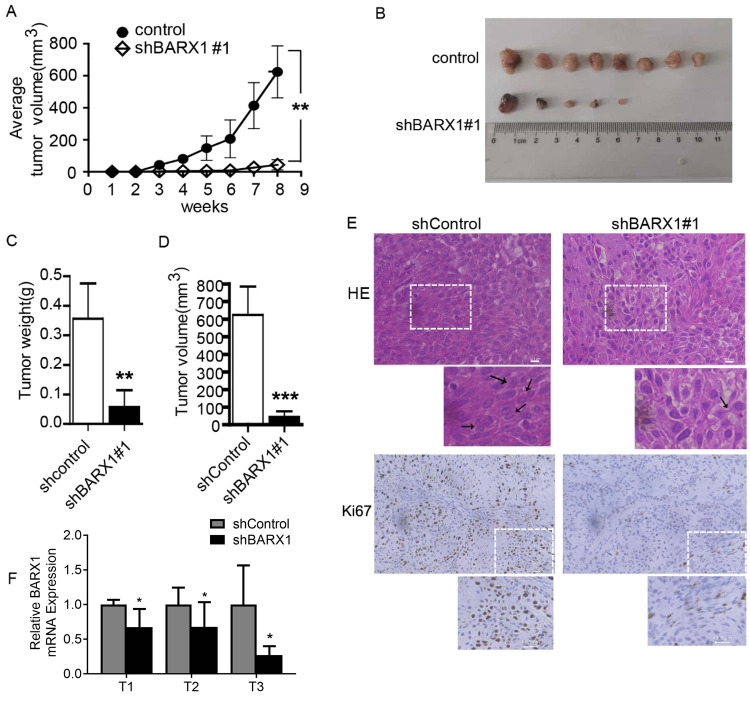
Silencing BARX1 inhibits lung cancer progression in vivo. **A** The change in tumor volume was determined every week after implantation. **B** Morphology of the tumor was photographed 8 weeks after inoculation. **C** Tumor weight and **D** tumor volume significantly decreased in the shBARX1 group compared with that of shControl. *n* = 8. **E** HE staining of the tumor tissues and the expression of Ki67 in each group was determined by IHC. Scale bar 12.5 µm. **F** The expression of BARX1 in tumor tissues was determined by RT-qPCR (*n* = 3).

To verify if BARX1 could promote lung cancer development and progression in vivo, nude mouse tumor xenograft was performed. The descrambled shRNA (ShControl) and *BARX1* shRNA (shBARX1#1) transfected A549 cells were subcutaneously inoculated into nude mice, respectively, followed by measuring tumor volume weekly. Eight weeks later, the mice were euthanized, and tumors were collected and photographed. We found that inoculation of shBARX1 transfected-A549 cells decreased 37% (3/8) tumor-formed rate in mice compared with inoculation of shControl A549 (Fig. 7A, B), accompanied by reducing tumor weight and volume (Fig. 7C, D), similar results were also obtained in the subcutaneous mouse model with H157 cells (Supplementary Fig. S4). Hematoxylin and eosin (H&E) staining (Fig. 7E, upper panel) showed that in the shControl grafted tumor, the tumor cells were tightly arranged and disordered, with relatively large nuclei, scanty cytoplasm and blurred boundary, and often with karyokinesis and cytokinesis (Fig. 7E, upper left panel). Unlike the shControl grafted tumor, the tumor cells derived from shBARX1 transfected-A549 were not close together, with a lower frequency of karyokinesis and cytokinesis (Fig. 7E, upper right panel).

The Ki67, a well-established proliferation marker, is present in all stages of the actively replicating cell nuclei and is used to determine tumor cell growth. Ki67 immunohistochemistry (IHC) staining (Fig. 7E, lower panel) showed that Ki67 expression was visibly decreased in the grafted tumor derived from shBARX1 transfected-A549 cells, compared to shControl (Fig. 7E, F). The results suggest that the knockdown of *BARX1* inhibits lung cancer progression in mice due to the downregulation of the downstream master oncogenes.

## Discussion

Most recent studies have revealed that BARX1 was highly expressed and played an oncogenic role in endometrial carcinoma (EC) [18] and clear cell renal cell carcinoma (ccRCC) [19] tissues. Differentially expressed TFs’ analysis showed that BARX1, as one of 10 potential oncogenic TFs, was required for NSCLC [20]. However, less is known about the cancer-promoting mechanism of BARX1, and the mechanism of BARX1 dysregulation during carcinogenesis has not been reported so far. Here, we have identified *BARX1* as a common DEG overexpressed in lung cancer LUSC and LUAD. Furthermore, BARX1 overexpression in LUSC and LUAD tissues from patients and in several NSCLC cell lines was validated. We showed that BARX1 overexpression promoted the proliferation, migration and invasion of NSCLC cells. Importantly, we have identified a novel mechanism for the regulation of *BARX1* gene expression, in which the RBP ZFP36 interacted with the 3′UTR of *BARX1* mRNA to mediate its destabilization. Subsequently, suppression of BARX1 by ZFP36 led to downregulation of the BARX1 downstream targets, thereby suppressing NSCLC cell proliferation, migration and invasion in vitro and tumorigenicity in mouse xenografts. These data provide new insights into the mechanism of BARX1 actions in NSCLC and aid a better understanding of the pathogenesis of NSCLC.

Not only malignant cells in the tumor core but also stromal infiltrating immune cells play a crucial role in tumor proliferation, invasion and metastasis. To delineate true residual signals representing individual cell populations, it is crucial to accurately estimate stroma-infiltrating immune cells and tumor purity (the fraction of cancer cells in a tumor) as well. We identified BARX1 as a key regulator involved in NSCLC progression by combining the ESTIMATE score with the transcriptome-based differently expressed genes analysis, and here mainly focused on its oncogenic role and regulation. Although a recent study showed that silencing BARX1 resulted in downregulated production of interleukins in allergic rhinitis-derived nasal fibroblasts, the exact role of BARX1 in tumor immune response remains to be elucidated [33].

In this study, we have demonstrated post-transcriptional control of *BARX1* gene expression by ZFP36. Eukaryotic gene control may occur at several steps, i.e., chromatin remodeling, transcription, post-transcription and translation. The intrinsic ability of the DNA sequence of a promoter region determines transcription activity. A study of genetic variants at 9q22 shows that the rs11789015-G allele markedly decreases the activity of the *BARX1* promoter with lower levels of *BARX1* mRNA and protein expression as compared with the A allele; however, there were no significant genotype-expression correlations for BARX1 expression in tumors and different genotypes of ESCC cell lines [17]. This means that there are certain unknown factors involved in *BARX1* dysregulation. Certain ARE-containing genes encode multiple cancer-associated factors that can promote cell growth, angiogenesis and invasion [22]. Sustained stabilization and enhanced translation of ARE-mRNAs are features of tumor cells, which is attributable to aberrant ZFP36-mediated post-transcriptional control of gene expression in cancers [23]. In general, the level of ZFP36 is in a dynamic balance under rigorous and precise control and responds to the external stimulus rapidly in vivo [22, 23]. In cancer, the change of ZFP36, such as expression levels, compartment localization and activity, will result in the overexpression of cancer ARE genes. The loss of RBP ZFP36 function leads to ARE-mRNA stabilization in a variety of human cancers such as prostate cancer, pancreatic cancer, breast cancer, colorectal cancer, malignant melanoma, hepatocellular carcinoma (HCC), malignant glioma, and lung cancer [22]. ZFP36-mediated post-transcriptional regulation of ARE genes has been demonstrated [22]. However, the functional interplay between ZFP36 and BARX1 is unknown.

In the present study, we showed the physic and functional interplay between the two molecules. ZFP36 could directly recognize and bound to the UAUU semi-binding site in the 3ʹUTR of *BARX1* mRNA to mediate *BARX1* mRNA destabilization, in which ARE-mediated mRNA decay, the main regulation mechanism of ZFP36 [22], might play a role. Upon this, the knockdown of ZFP36 could increase, but overexpression of ZFP36 decreases BARX1 expression in NSCLC cells. We also showed that BARX1 was overexpressed and ZFP36 was downregulated in NSCLC tissues of patients. Based upon the abovementioned physic and functional interplay between ZFP36 and BARX1 in lung cancer tissues and NSCLC cell lines, we conclude that ZFP36 plays a suppressor role in NSCLC development by limiting the unscheduled accumulation of *BARX1* mRNA, and the dysregulation of BARX1 during carcinogenesis is presumably attributable to the loss of ZFP36. The functional interplay was further confirmed by the in vivo tumor xenograft experiment (Supplementary Fig. S5A–E), which showed that BARX1 overexpression promoted tumor progression, and importantly, ZFP36 significantly attenuates BARX1-mediated tumor progression in vivo. However, our data does not exclude the possibilities of the involvement of others in the dysregulation of BARX1 in NSCLC tissues, such as aberrant chromatin remodeling and transcriptional activation, and somatic gene mutation. Furthermore, the reason for ZFP36 loss or defect in NSCLC development is not clear, which needs further exploration in the future.

Several BARX1-related studies focus on its role in the regulation of certain organ formation and development, in which BARX1 acts to regulate the expression of signaling molecules [15–18]. For instance, Barx1 regulates the expression of Wnt antagonists Sfrp1 and Sfrp2 to attenuate Wnt signaling, allowing digestive tract endoderm to differentiate into highly specialized stomach epithelium [34]. The attractive attention of the BARX1 oncogenic role has begun only in recent years, based on the identification of DEGs from tumor tissues and matched normal adjacent tissues [17–20]. However, the mechanism for BARX1 action in carcinogenesis is poorly understood. We found that BARX1 bound to the promoters of *CDC20*, *CDC45*, *TRIM37* and *MMP-9* genes to activate their expression, and knockdown of BARX1 significantly decreased their mRNA levels. We therefore conclude that BARX1 transactivates *CDC20*, *CDC45*, *TRIM37* and *MMP-*9 genes, thereby promoting cellular proliferation, migration and invasion of lung cancer cells due to the functional roles of these genes in cell-cycle progression, DNA synthesis and their association with carcinogenesis.

Cell division cycle 20 (CDC20) is a component of cell division and is responsible for anaphase initiation by binding and activating the anaphase-promoting complex/cyclosome (APC/C), an E3 ubiquitin ligase complex, to modulate mitotic exit through the degradation of various critical cell-cycle regulators [35, 36]. A recent study has shown that overexpression of CDC20 promoted the metastasizing capacities of pancreatic cancer cells and breast cancer cells [37]. Abnormal expression of CDC20 is commonly associated with malignant progression and poor prognosis in various types of cancer [36, 38]. Cdc45 is a key factor in the transition from G1 phase to S phase: in the early S-phase, CDC45, together with MCM2-7 and GINS, forms the replicative helicase CDC45-MCM2-7-GINS (CMG) complex for initiating DNA synthesis [39, 40]. CDC45 is upregulated in various human carcinomas, leukemia, and lymphoma [41]. Bioinformatic analysis of functional hub genes in NSCLC showed CDC45 as an oncogene linking to prognosis of NSCLC patients [42, 43]. Tripartite motif-containing 37 (TRIM37), a member of the TRIM family, is an oncogenic H2A ubiquitin ligase that is overexpressed in a subset of breast cancers and promotes transformation by facilitating the silencing of tumor suppressors [44]. Consistently, the knockdown of TRIM37 reduces tumorigenicity in mouse xenografts [44, 45]. It is well known that MMP-9 mainly degrades gelatin, collagens IV and V in ECM and basement membrane through its proteolytic function. MMP9 is implicated in cancer development and progression through its activities in cell apoptosis, proliferation, and angiogenesis. In particular, MMP-9 is pivotal in many steps of the metastatic process [46, 47]. Considering the functional characteristics of CDC20, CDC45, TRIM37 and MMP9, it is not surprising that BARX1 promotes cellular proliferation, migration and invasion of lung cancer cells by transactivating *CDC20*, *CDC45*, *TRIM37* and *MMP-*9 expression.

**Fig. 8:**
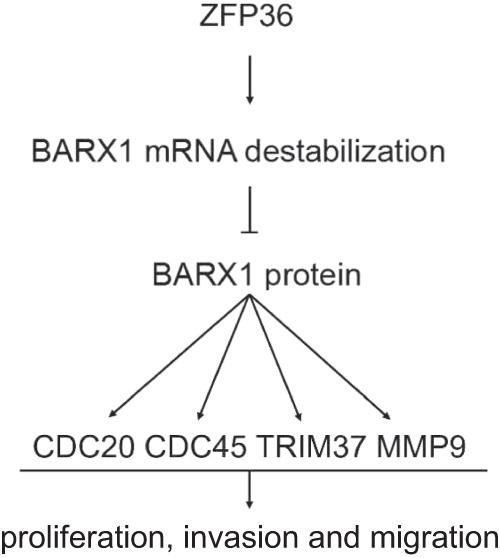
Schematic diagram summarizing the role of ZFP36 on regulating BARX1 in NSCLC. The RNA-binding protein ZFP36 destabilizes *BARX1* mRNA and downregulates the expression of BARX1. Decreased BARX1 reduces the expression levels of MMP-9, CDC20, CDC45 and TRIM37, inhibits the proliferation, migration and invasion of lung cancer cells.

Personalized medicine has been defined as “a form of medicine that uses information about a person’s genes, proteins and environment to prevent, diagnose and treat disease”. To realize cancer precision medicine, scientists and physicians devote themselves to defining specific genetic alterations (e.g., mutation, rearrangement, amplification, and so on) and abnormal gene expression patterns (i.e., DEG) as biomarkers that match with particular cancer-targeted therapy. Based on the LUAD and LUSC RNA-seq data from TCGA and ESTIMATE databases, we identified BARX1 as an oncogenic TF and DEG commonly expressed in NSCLC, and its dysregulation was verified in NSCLC patients and cell lines. Since the most direct effect on gene expression is exerted by transcriptional factors, it is not surprising that following the increased expression of BARX1, its targets were upregulated, while the RBP ZFP36 was downregulated (Fig. 8). Conceivably, BARX1, together with CDC20, CDC45, TRIM37, MMP plus ZFP36 may have potential diagnosis and prognosis values in NSCLC, although these data were limited by the small sample size due to that we excluded patients who had received chemotherapy or radiotherapy. Another limitation of our study is the limited number of LUSC cases, which tallies its lower clinical incidence than LUAD.

In summary, we have identified BARX1 as an oncogenic transcriptional factor that was highly expressed in NSCLC tissues and cell lines. We found that dysregulation of BARX1 in NSCLC was attributable to the loss of RBP ZFP36 that bound to the 3’UTR of *BARX1* mRNA to mediate its destabilization. Furthermore, high-expressed BARX1 transactivated its downstream genes *CDC20*, *CDC45*, *TRIM37* and *MMP-9*, and ultimately promoted growth, proliferation, migration and invasion of NSCLC cells. These data may provide further insights into the mechanism of BARX1 actions in NSCLC and aid a better understanding of the pathogenesis of NSCLC and develop potential prognostic biomarkers or therapeutic targets for NSCLC.

## Materials and methods

### Patient and tissue samples

The study protocol was approved by the Tsinghua University Changgung Hospital Research Ethics Board (No 20281-0-02). A total of 23 NSCLC cases who underwent curative surgery, without prior treatments, at the Changgung Hospital of Tsinghua University (Beijing, China) from September 2020 to October 2021 were enrolled in this study. All the cases had no history of other tumors by examination of a plain chest radiograph, CT scan, and bone scan and were diagnosed with NSCLC by at least two pathologists. The patients’ medical records were reviewed to obtain data, including age at diagnosis, sex, nationality, and smoking history (Supplementary Table 1). The tumor tissues and matched normal adjacent tissue specimens were collected at the time of surgery and quickly frozen in liquid nitrogen.

### Genome-scale bioinformatic analysis

The RNA-seq data for LUAD, LUSC and normal lung tissues, clinical information of LUAD and LUSC patients, were downloaded from The Cancer Genome Atlas (TCGA) database via Genomic Data Commons Data Portal. The immune score, stromal score and estimate score of LUAD samples were obtained through ESTIMATE (Estimation of Stromal and Immune cells in Malignant Tumor tissues using Expression data) (https://bioinformatics.mdanderson.org/estimate/). DAVID (Database for Annotation, Visualization and Integrated Discovery, http://david.abcc.ncifcrf.gov/) was used to explore the GO functional and KEGG pathway enrichment analysis. The correlation between BARX1 and ZFP36 expression was analyzed by correlation analysis of the Spearman method for 969 lung cancer samples and 685 normal samples from the TCGA database, GTEx database and Cancer Cell Line Encyclopedia databases (CCLE). ZFP36 binding sites in the 3’UTR of *BARX1* mRNA were determined with the information in the NCBI database. DNA binding domain, protein structure and downstream genes of BARX1 were analyzed through the JASPAR database (http://jaspar.genereg.net/).

### Cell culture

Human NSCLC cell lines A549, GLC-82, H1975, H157, and human bronchial epithelial cell lines BEAS-2B and HBEC cells were cultured in DMEM medium (GIBICO, BRL, CA, USA) supplemented with 10% fetal bovine serum (Zeta-Life, CA, USA) at 37 °C with 5% CO_2_.

### Constructs and lentivirus packaging

shRNAs were designed to specifically target ZFP36 and *BARX1* mRNA using BLOCK-iT™ RNAi Designer (http://rnaidesigner.thermofisher.com/rnaiexpress/). The oligos were synthesized by Generay Company (Shanghai, China). The oligos were annealed and inserted into the pLKO.1 lentiviral vector (LV) at EcoRI and AgeI sites to generate pLKO.1-shBARX1 and pLKO.1-shZFP36, respectively. Lentivirus packaging was performed, and target cells were infected. The pcDNA3.1-3×Flag-ZFP36 expression vector was constructed by inserting ZFP36 cDNA into the BamHI and XhoI sites. The oligo sequences are listed in Supplementary Table 2.

### Transfection and LV infection of cells

Cells were transiently transfected with small interfering RNA (siRNA) or pcDNA3.1-3×Flag-ZFP36 expression plasmid using lipofectamine**®** 2000 or lipofectamine® RNAiMAX Transfection Reagent (Invitrogen). To generate a stable cell line expressing shRNA, cells were infected by the HEK293T-packed lentiviral particles carrying pLKO.1-shBARX1 or pLKO.1-shZFP36 vectors using FuGENE6 (Promega) according to the manufacturer’s instructions. The infected cells were then selected by puromycin and expanded. ZFP36 and BARX1 expression levels in the selected clones were determined by RT-qPCR and Western blotting.

### RNA isolation and real-time quantitative PCR (RT-qPCR)

Total RNA was extracted using Trizol (Invitrogen, MA, USA) according to the manufacturer’s instructions. For mRNA detection, 2 μg total RNA was subjected to reverse transcription using Reverse Transcription Kit (Thermo Scientific, MA, USA). PCR amplification was performed with the primers. Each PCR mixture contained 2 µl cDNA, 200 nM of each primer and 11 µl of the Power SYBR Green PCR Master Mix (TOYOBO) in a 20 µl reaction mixture. RT-qPCR was performed using ABIPRISM 7500 Real-Time PCR system (Applied Biosystems). Data were normalized to GAPDH according to the manufacturer’s protocol. The primer sequences are listed in Supplementary Table 2.

### Western blotting

Total protein was extracted using radioimmunoprecipitation assay (RIPA) buffer, and protein concentration was determined using BCA Protein Assay Kit (Thermo Scientific). Thirty micrograms of total protein was subjected to 10% SDS PAGE, transferred onto nitrocellulose membranes, and probed with specific antibodies against BARX1 (ab181851, Abcam), ZFP36 (ABE285, Millipore Sigma) or ɑ-Tubulin (PM054, MB). Proteins on the membrane were immunostained with aIRDye 800CW secondary antibody (IgG) for 1 h. Blots were visualized with the LI-COR Odyssey image analysis system (Li-cor Biosciences).

### Luciferase reporter constructs and luciferase assay

To construct the luciferase reporter plasmids, a 537-bp 3’UTR of *BARX1* mRNA, bearing the wild-type or mutant binding site of ZFP36, was acquired using PCR and inserted into the pMIR-Report plasmid (Applied Biosystems) at the Bmt I and Xba I sites to generate pMIR-Report-BARX1-3’ UTR-WT (wild type) and pMIR-Report-BARX1-3’ UTR-MuT (mutant). Luciferase activity was detected using the Dual Luciferase Assay (Promega) according to the manufacturer’s instructions. Then, 48 h after transfection, the cells were harvested and lysed, the *firefly* and *renilla* luciferase activities were assayed with the Dual-Luciferase Reporter System (Promega, Madison, WI, USA). The primer sequences are listed in Supplementary Table 2.

### Cell proliferation assay

Cells were seeded onto 96-well plates at the density of 2000 cells per well. Each well contained 90 µl DMEM and 10 µl Cell Counting Kit 8 (Jude Antai Technology). After cultured for 2 h at 37 °C, the absorbance value of each well was measured at 450 nm. The data were collected for 24, 48 and 72 h, and each experiment was performed in triplicate.

### Colony formation assay

Cells were plated onto six-well plates with 500 cells per well and incubated in a 37 °C incubator for 10 days. Colonies were fixed in 4% paraformaldehyde for 15 min and stained with 0.1% Crystal Violet Staining solution for 20 min and counted using a microscope at 40× magnification.

### Wound healing assay

For cell migration and invasion assays, the transfected cells were seeded onto six-well plates and cultured overnight. Wounds were created by scratching the cell layer with a sterile 200 µl plastic pipette tip and washed with culture medium. Cells were further cultured with medium containing 1% FBS for 24 h, 48 h and 72 h. Images were acquired by microscopy (Leika) at 100× magnification.

### Transwell

Cell invasion was evaluated by Corning® BioCoat™ Matrigel® Invasion Chamber (Transwell). The cells were cultured at 37 °C for 48 h before seeding. The chamber was placed in a 24-well plate, and a complete medium containing 1 × 10^5^ cells was added to the top chambers. After incubation for 24 h, the chamber was moved out and washed with PBS twice, followed by fixation in 4% paraformaldehyde for 15 min after the removal of Matrigel. The invading cells were stained with 0.1% crystal violet and counted under the microscope (Leica). The number of transmembrane cells was calculated by the Image J system.

### RNA immunoprecipitation (RIP)

Briefly, cell lysate was prepared in a lysis buffer containing a protease inhibitor cocktail and RNase inhibitor. Then, protein A/G magnetic beads were prepared for incubation with 5 μg of purified antibodies per immunoprecipitation reaction. Further, the mixture was incubated with rotation overnight at 4 °C to precipitate RNA-binding protein-RNA complexes. Finally, RNA was purified using proteinase K buffer and examined by quantitative reverse transcription polymerase chain reaction (qRT-PCR).

### RNA pull-down assay

RNA pull-down assay was performed using Pierce™ Magnetic RNA-Protein Pull-Down Kit (20164, Thermo Scientific) according to the manufacturer’s instructions. Briefly, biotin-labeled RNA probes, including wild-type and mutant probes, were incubated with streptavidin magnetic beads for 3 h at room temperature. The lysates of the cells were then incubated overnight at 4 °C with streptavidin magnetic beads. Proteins bound to magnetic beads were eluted and examined using Western blotting.

### Chromatin immunoprecipitation-qPCR (ChIP-qPCR)

A549 cells were cross-linked with 1% formaldehyde at RT for 10 min. The cross-linked chromatin was sonicated to generate DNA fragments averaging 100–200 bp in length by Bioruptor plus. Chromatin fragments were immunoprecipitated with antibodies against mouse normal IgG (4 μg, Santa Cruz), BARX1 (4 μg, Santa Cruz) and protein A-Sepharose beads. After washing and reversing the cross-links, the enriched DNA was purified and then examined by qRT-PCR. The primer sequences are listed in Supplementary Table 2.

### RNA half-life analysis

To analyze the half-life of *BARX1* mRNA, A549 cells were transfected with the pcDNA3-ZFP36 plasmid at 70–80% cell confluence. Then, 48 h after transfection, cells were treated with actinomycin D (Sigma-Aldrich, Saint Louis, USA) for 4, 2 and 0 h. Then cells were harvested, and RNA was extracted. The mRNA levels at different times were analyzed by RT-qPCR.

### Mouse tumor xenografts

Six-week-old nude mice were randomly grouped. The control shRNA (ShControl) or *BARX1* shRNA (shBARX1) transfected A549 cells were subcutaneously inoculated into the right axilla of each nude mouse. The tumor volume was monitored each week after inoculation. The tumor volumes were calculated according to the formula (L x W^2^)/2. Eight weeks later, the mice were euthanized, and tumors were collected and photographed. The study was approved by the ethics committee of Peking University Health Science Center for animal research, and all animal experiments conformed to the Guide for the Care and Use of Laboratory Animals of the Health Science Center of Peking University.

### Immunohistochemistry (IHC) and histochemistry staining

Mouse xenograft tumor samples were fixed in 10% formalin for 24 h, embedded in paraffin and cut into 4-µm sections. Slices were immunostained at 4 °C overnight with primary antibodies against Ki67 (GB111141, servicebio, 1:800), followed by incubation with FITC-conjugated goat anti-rabbit antibody (GB23303, servicebio,1:200) for 1 h at room temperature. For hematoxylin and eosin (H&E) staining, 4-µm paraffin tissue sections were deparaffinized, rehydrated, and stained with hematoxylin (G1003, servicebio, Beijing, China) for 3 min, after treated with 1% acid ethanol and rinsed in distilled water, the sections were stained with eosin (G1003, Servicebio, Beijing, China) solution for 5 min and followed by dehydration with graded alcohol and clearing in xylene. Finally, the slices were observed and photographed under a microscope (Leica Microsystems, Hessen, Germany). Immunostaining sections were analyzed using Image-pro plus processing system.

### Statistical analysis

The SPSS version 18 for Windows was used for statistical analysis. Continuous variables were expressed as mean ± SD. The Student’s *t*-test and Wilcoxon’s rank-sum test were used for statistical analysis. Spearman correlation was calculated between the expression levels of ZFP36 and BARX1 in LUAD. Statistical significance was defined by a two-tailed *p*-value of 0.05.

## Supplementary information

Supplementary data

Supplementary file 1

Supplementary file 2

Response to the author list changes

Original Data File

## Data Availability

The data supporting this study are available on request from the corresponding author.
