# Correction: ZFP36 loss-mediated BARX1 stabilization promotes malignant phenotypes by transactivating master oncogenes in NSCLC

**DOI:** 10.1038/s41419-025-07718-6

**Published:** 2025-05-22

**Authors:** Tongjia Zhang, Lizhen Qiu, Jiashun Cao, Qiu Li, Lifan Zhang, Guoshun An, Juhua Ni, Hongti Jia, Shuyan Li, Kailong Li

**Affiliations:** 1 Department of Biochemistry and Biophysics, Beijing Key Laboratory of Protein Posttranslational Modifications and Cell Function, School of Basic Medical Sciences, Peking University Health Science Center, 100191 Beijing, China; 2 Department of Thoracic Surgery, Beijing Tsinghua Changgung Hospital, School of Clinical Medicine, Tsinghua University, 102218 Beijing, China; 3 Department of Research, Beijing Tsinghua Changgung Hospital, School of Clinical Medicine, Tsinghua University, 102218 Beijing, China

**Keywords:** Non-small-cell lung cancer, Oncogenesis

Correction to: *Cell Death and Disease* 10.1038/s41419-023-06044-z, published online 16 August 2023

We noted that the original version of this article contained an error. Specifically, the image for si-BARX1#2 in Fig. 5D was erroneously incorporated during the compilation of graphical figures. However, this mistake doesn’t affect the conclusion in this study.

**Amended Fig 5** 
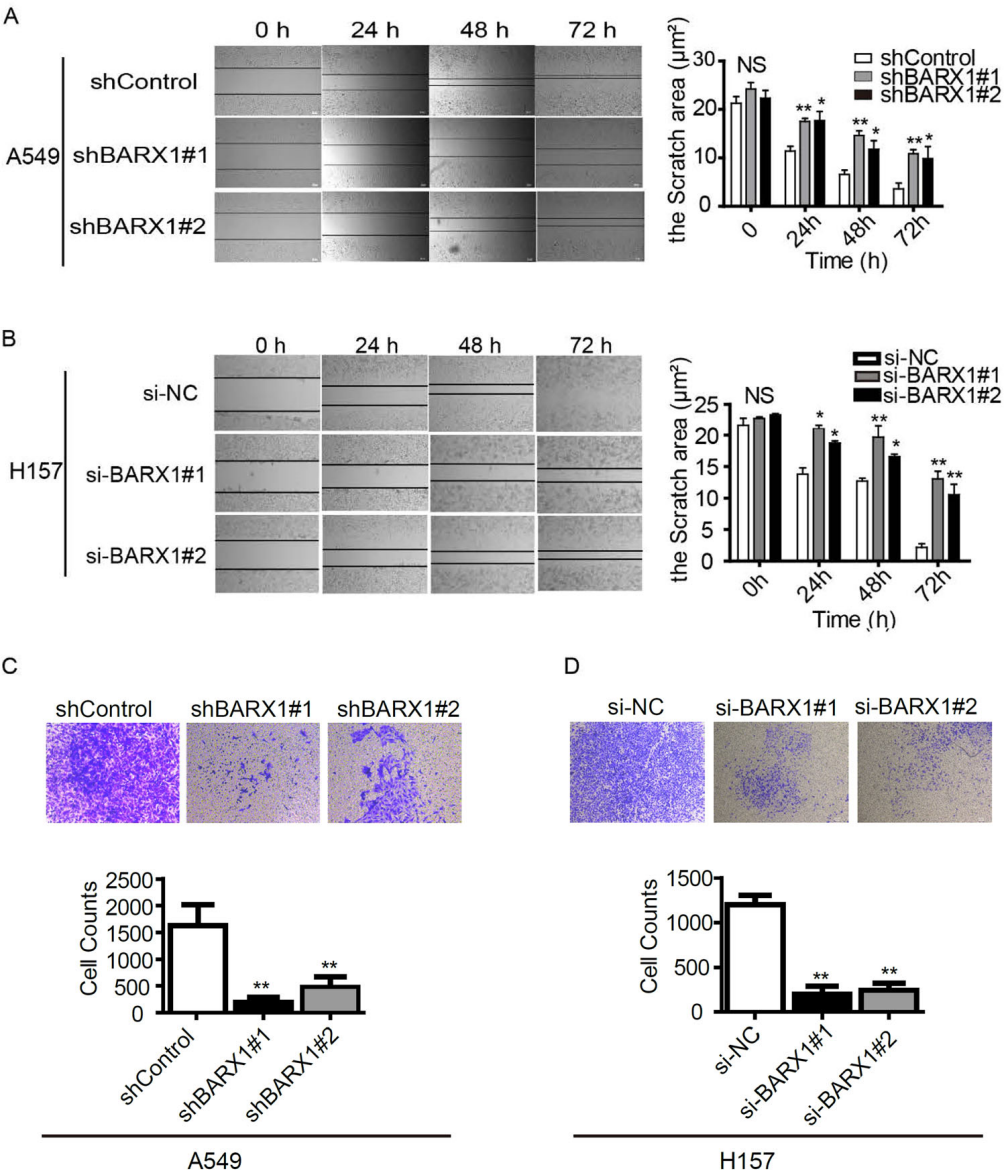


**Original Fig 5** 
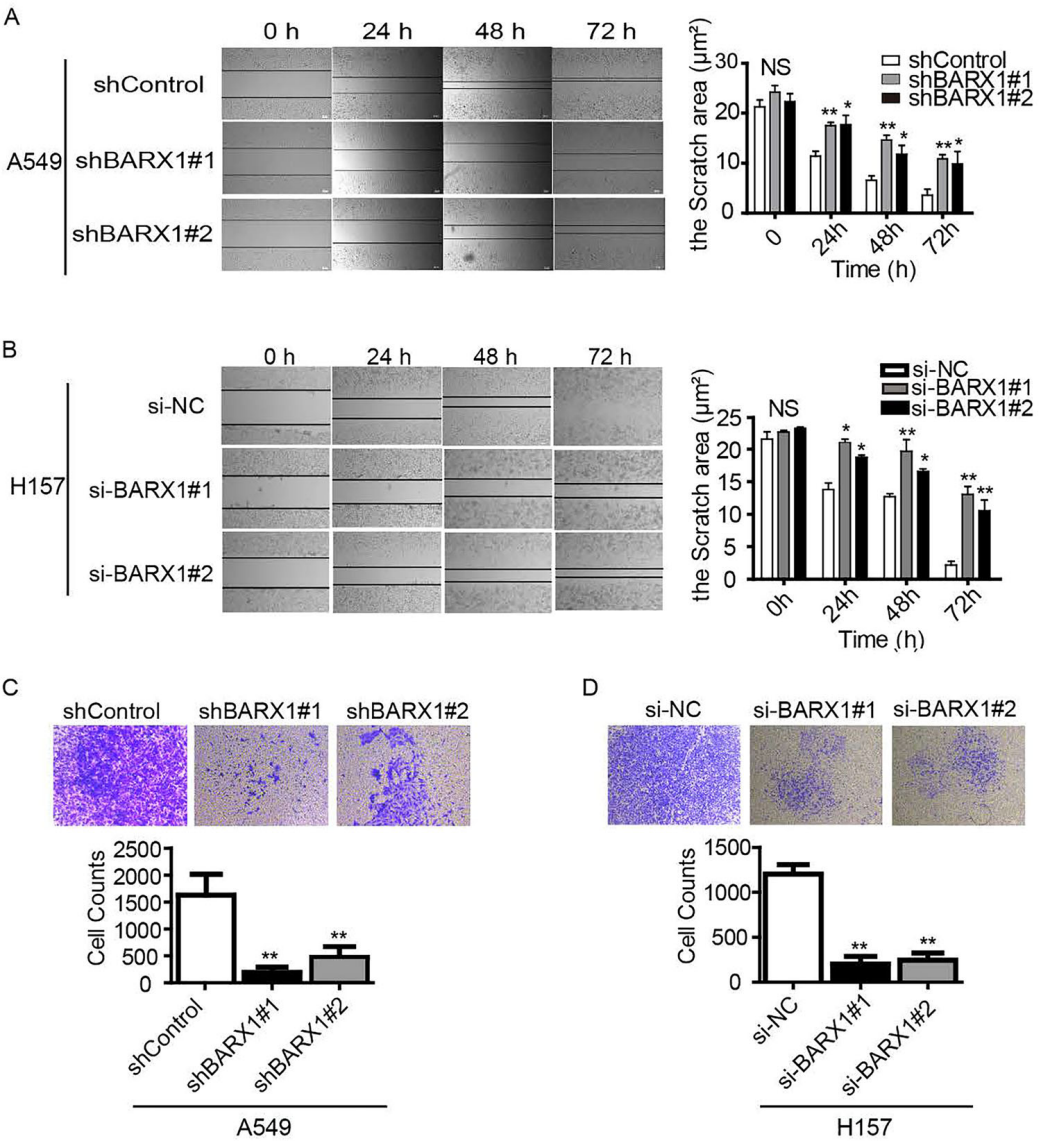


**Amended Fig 5D** 
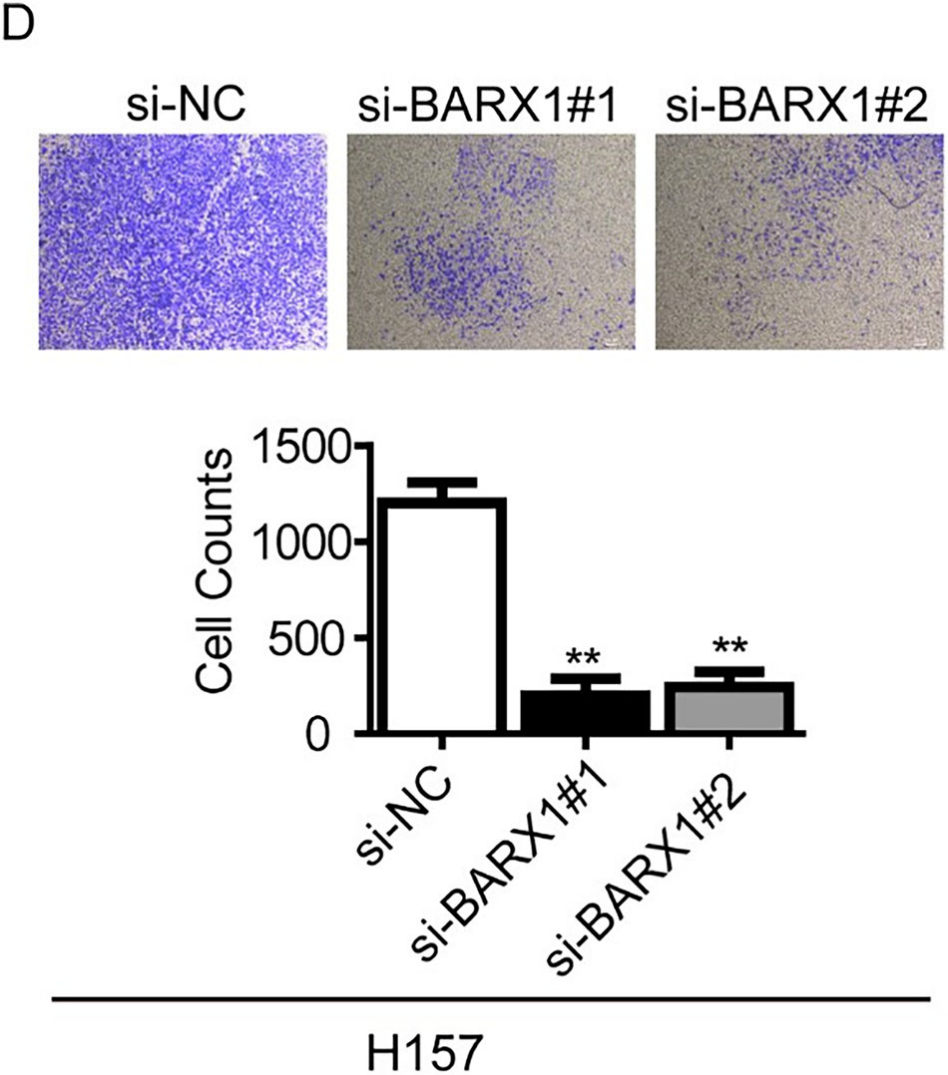


**Original Fig 5D** 
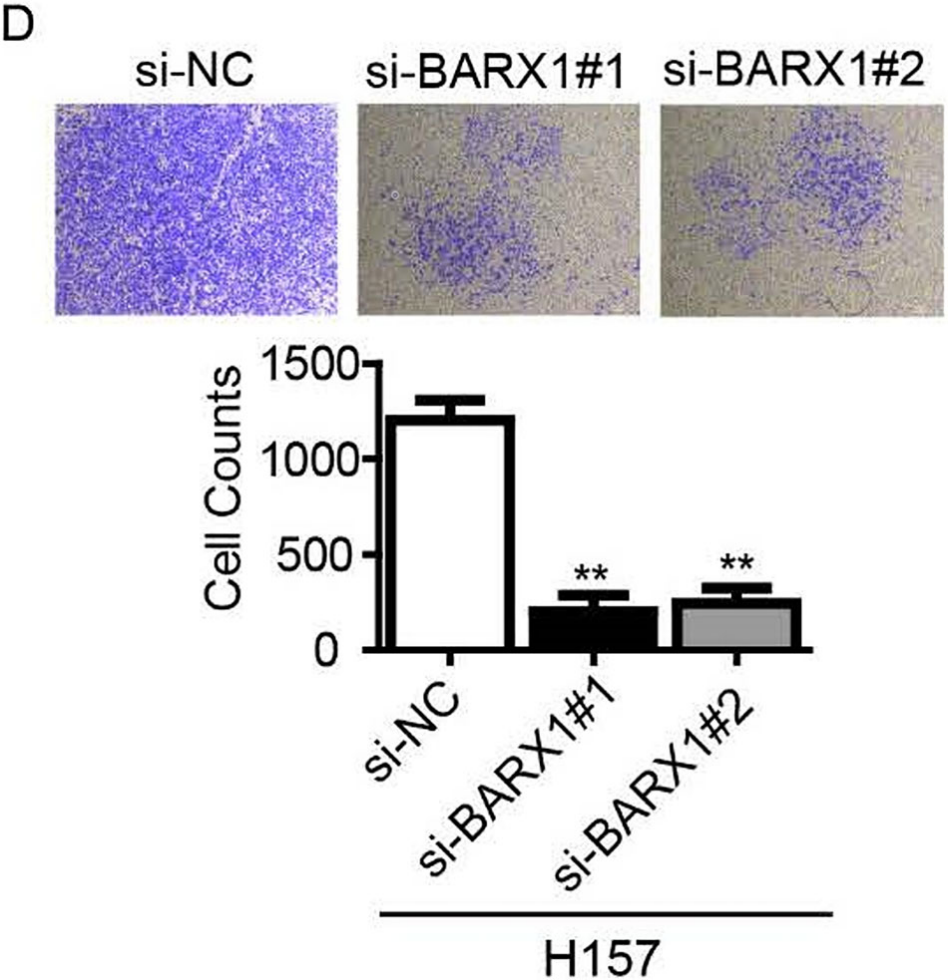


The original article has been corrected.

