# Supplementary material for: ZFP36 loss-mediated BARX1 stabilization promotes malignant phenotypes by transactivating master oncogenes in NSCLC
Source: Cell Death Dis. 2023 Aug 16;14(8):527. doi: 10.1038/s41419-023-06044-z (PMC10432398; doi:10.1038/s41419-023-06044-z)
Supplement: Supplementary file 1 — Supplementary data [file 41419_2023_6044_MOESM1_ESM.pdf]

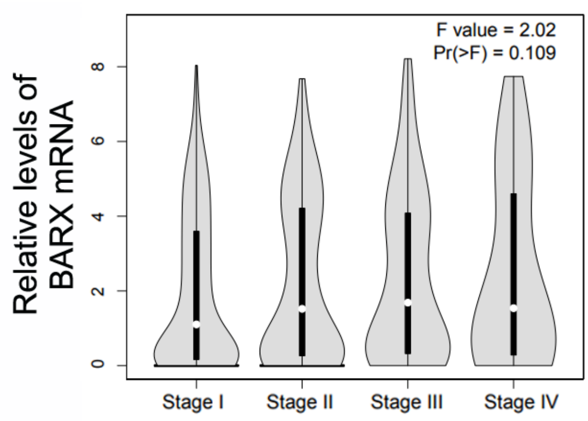


**Figure S1. Analysis of BARX1 expression in LUAD and LUSC samples.**

Comparison of BARX1 mRNA level in different stages of the LUAD and LUSC tissues. F statistic value and p value associated with the F statistic were shown upright.

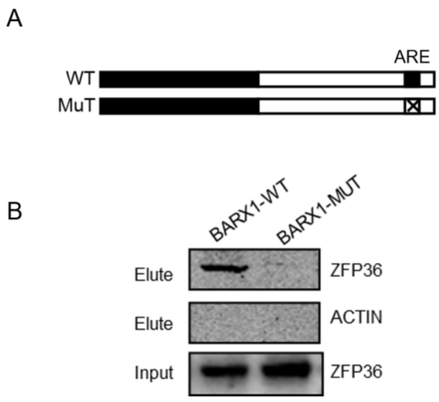


**Figure S2. Specific interaction of ZFP36 with the 3'-UTR sequence of BARX1.**

(A) Construction of wild-type and mutant probe. (B)The effect of mutation on the ability of 3'-UTR sequence of BARX1 to bind ZFP36 was evaluated by the RNA-Protein pull down assay. Wild-type and mutant 3'-UTR sequence of BARX1 were labeled with biotin and used for the pull-down assay. Samples were normalized by volume, and ACTIN was used as a negative control.

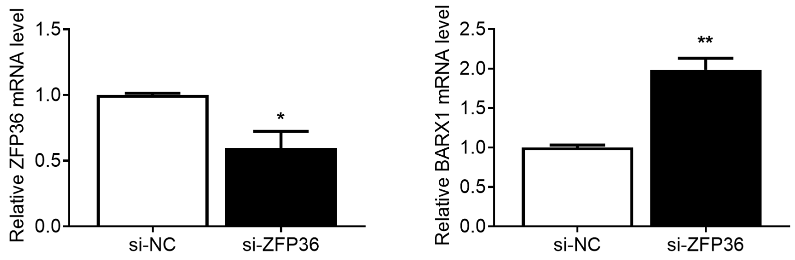


**Figure S3. Silencing of ZFP36 negatively regulates BARX1 expression.**

Upregulation of BARX1 expression (right panel) induced by ZFP36 knockdown (left panel) in A549 cells was verified by RT-qPCR, (n=3, *p<0.05, **p<0.01).

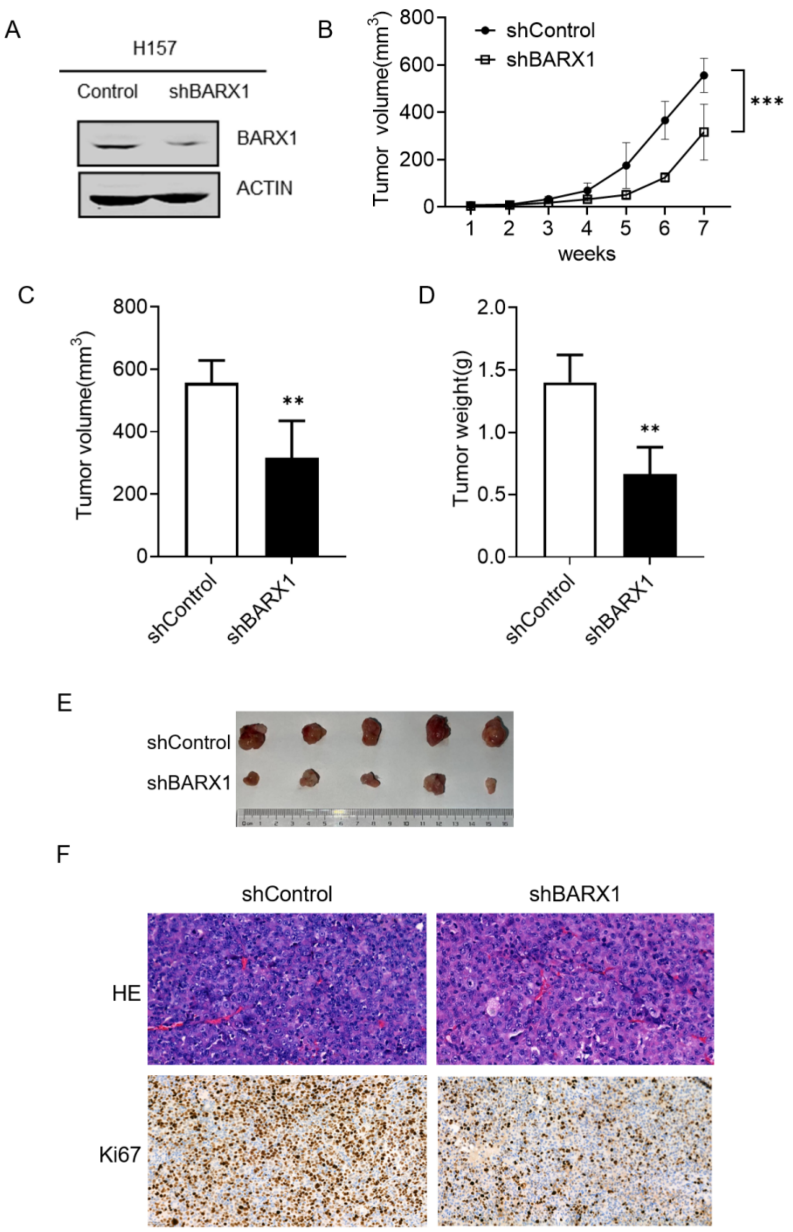


**Figure S4. Silencing BARX1 inhibits tumor progression in H157 cells in vivo.**

(A) Knockdown of BARX1 in H157 cells was verified by Western blot. (B) The change in tumor volume was determined every week during the tumor growth after tumor cells implanted. (C) Tumor volume and (D) weight of the mice were significantly decreased in shBARX1 group compared with that of shControl (n=5, p**<0.01, p***<0.001). (E) Morphology of tumor was photographed seven weeks after inoculation. (F) HE and immunohistochemical staining of the shControl and shBARX1 group tumor tissues, the expression of Ki67 of tumor sections were determined by IHC.

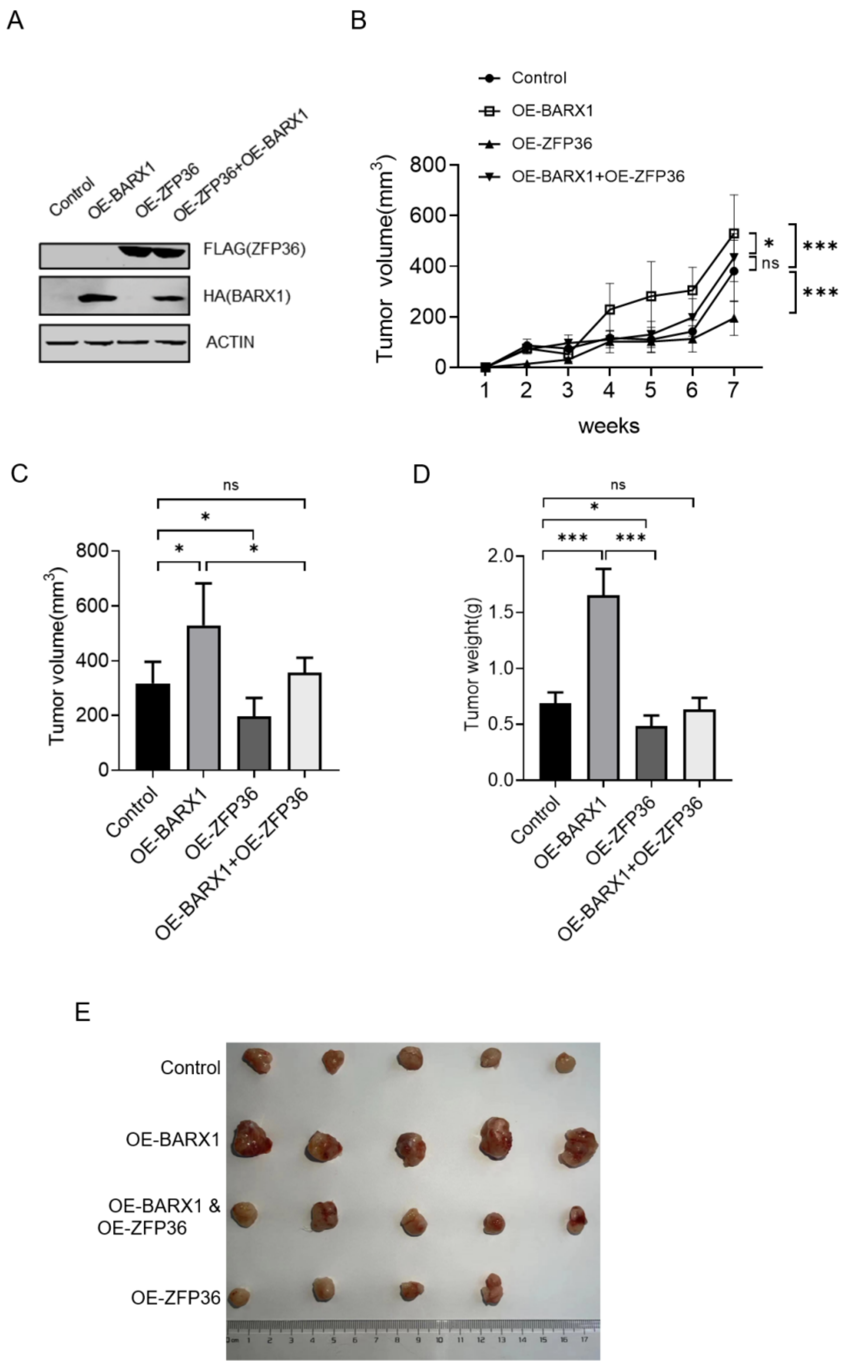


**Figure S5.** **ZFP36 attenuates BARX1-medaited tumor progression in vivo.**

(A) Western blot was performed to verify the overexpression of BARX1 and/or ZFP36 in A549 cells. BARX1 was fused with HA tag, and ZFP36 was fused with FLAG tag. OE-BARX1: BARX1 overexpression; OE-ZFP36: ZFP36 overexpression; OE-BARX1+ OE-ZFP36: BARX1 and ZFP36 co-overexpression. (B) The change in tumor volume was determined every week during the tumor growth after tumor cells implanted (n≥4, p*<0.05, p***<0.001). (C) Tumor volume and (D) weight of the mice were significantly attenuated in OE-BARX1+ OE-ZFP36 group compared with that of OE-BARX1. (E) Morphology of tumor was photographed seven weeks after inoculation.

**Supplementary Table 1. The clinical information of patients.**

| Characteristics | Number |
|---|---|
| age | |
| >60 | 16 |
| <60 | 7 |
| Pathological_stage | |
| IA | 9 |
| IB | 6 |
| IIA | 1 |
| IIB | 2 |
| IIIA | 4 |
| IVA | 1 |
| Sex | |
| male | 13 |
| female | 10 |
| T | |
| T1a | 3 |
| T1b | 4 |
| T1c | 3 |
| T2a | 8 |
| T2b | 1 |
| T3a | 2 |
| T4 | 2 |
| Postperative diagnosis | |
| LUAD | 19 |
| LUSC | 4 |

**Supplementary Table 2. Primers used in this study.**

| Name | Sequences | Application |
|---|---|---|
| CDC45-F | CAACAGTGTTTGCGTTCG | ChIP-qPCR |
| CDC45-R | GCTTCTTAGTTCCCAGCC | ChIP-qPCR |
| TRIM37-F | AGCATCTTGCCAACTAAAT | ChIP-qPCR |
| TRIM37-R | AGTTAAGATTCTTGTATG | ChIP-qPCR |
| MMP-9-F | AGGATATCTGACCTGGGA | ChIP-qPCR |
| MMP-9-R | CTTGTGGGAACTGTATGA | ChIP-qPCR |
| CDC20-F | ACCTCTAACCTAAGCATAT | ChIP-qPCR |
| CDC20-R | GTCATTAGCTTAAAGTCCACT | ChIP-qPCR |
| shBARX1-1 | GCCGGACAGAATAGATCTTGC | shRNA |
| shBARX1-2 | CCTGACAGAATAGATCTAGCT | shRNA |
| shZFP36-1 | GATCCGACCCTGATGAATATG | shRNA |
| shZFP36-2 | GCGCTACAAGACTGAGCTATG | shRNA |
| GAPDH-F | TGTCAGTGGTGGACCTGACCT | qPCR |
| GAPDH-R | AGGGGAGATTCAGTGTGGTG | qPCR |
| BARX1-F | TTCCACGCCGGACAGAATAG | qPCR |
| BARX1-R | CTGCTCGCTCGTTGGAATTG | qPCR |
| ZFP36-F | CGCTACAAGACTGAGCTATG | qPCR |
| ZFP36-R | CCTGGAGGTAGAACTTGTG | qPCR |
| MMP-9-F | GTGCTGGGCTGCTTTGCTG | qPCR |
| MMP-9-R | GTCGCCCTCAAAGGTTTGGAAT | qPCR |
| CDC20-F | GCACAGTTCGCGTTCGAGA | qPCR |
| CDC20-R | CTGGATTTGCCAGGAGTTCGG | qPCR |
| CDC45-F | TTCGTGTCCGATTTCCGCAAA | qPCR |
| CDC45-R | TGGAACCAGCGTATATTGCAC | qPCR |
| TRIM37-F | TATGGAGAAATTGCGGGATGC | qPCR |
| TRIM37-R | GTCAGCCAGCGCCTAATACAG | qPCR |
| BARX1-WT-F | GCTAGCGGGCGGTATACGGTG | Cloning |
| BARX1-WT-R | TCTAGATTGTGTCATAAAATA | Cloning |
| BARX1-MT-F | GCTAGCGGGCGGTATACGGTGC | Cloning |
| BARX1-MT-R | TCTAGATTGTGTCATAAGGAG | Cloning |
