# Supplementary material for: ZFP36 loss-mediated BARX1 stabilization promotes malignant phenotypes by transactivating master oncogenes in NSCLC
Source: Cell Death Dis. 2023 Aug 16;14(8):527. doi: 10.1038/s41419-023-06044-z (PMC10432398; doi:10.1038/s41419-023-06044-z)
Supplement: Supplementary file 4 — Response to the author list changes [file 41419_2023_6044_MOESM4_ESM.pdf]

BARX1 story: Accepted in Principle

New Meeting
2023-08-04 21:19:40

From : "kailongli" <kailongli@pku.edu.cn>

To : 673048571@qq.com lizhenqiu0808@163.com cjsa01253@btch.edu.cn lqa02099@btch.edu.cn lfzya1@163.com guoshunan@bjmu.edu.cn juhuani@bjmu.edu.cn jiahongti@bjmu.edu.cn shuyanli@bjmu.edu.cn [Hide details]

Sent Successfully. [Show detail] To the recipient of 9, among them 9 is Successfully delivered to mail server

Hi all,

Our manuscript entitled "ZFP36 loss mediated BARX1 stabilization promotes malignant phenotypes by transactivating master oncogenes in NSCLC" has been provisionally accepted for publication in *Cell Death & Disease*. Here is the last thing before official acceptance.

Our most recent author list differs from the one in the original submission because of the revision. To move forward, please **reply "Agree" to my email** confirming that you agree to these changes. Thanks.

The current list is:

Tongjia Zhang1,4, Lizhen Qiu1,4, Jiashun Cao2,4, Qiu Li3,4, Lifan Zhang1,4, Guoshun An1 , Juhua Ni1, Hongti Jia1, Shuyan Li1 *, Kailong Li1 *

Best,

Kailong

1. Tongjia Zhang

回复：BARX1 story: Accepted in Principle

From : "我知道" <673048571@qq.com>

To : "kailongli" <kailongli@pku.edu.cn>

Agree

2. Lizhen Qiu

Re: BARX1 story: Accepted in Principle

From : "lizhenqiu0808" <lizhenqiu0808@163.com>

To : "kailongli" <kailongli@pku.edu.cn>

Agree

3. Jiashun Cao

Re: BARX1 story: Accepted in Principle

From : "曹加顺" <cjsa01253@btch.edu.cn>

To : "lishuyan" <shuyanli@bjmu.edu.cn> "kailongli" <kailongli@pku.edu.cn>

Cc : "673048571" <673048571@qq.com> "lizhenqiu0808" <lizhenqiu0808@163

Agree

4. Qiu Li

Re: BARX1 story: Accepted in Principle

From : "李秋" <lqa02099@btch.edu.cn>

To : "曹加顺" <cjsa01253@btch.edu.cn> "lishuyan" <shuyanli@bjmu.edu.cn> "kailongli" <kailongli@pku.edu.cn>

Cc : "673048571" <673048571@qq.com> "lizhenqiu0808" <lizhenqiu0808@163.com> "lfzya1" <lfzya1@163.com>

Agree

5． Lifan Zhang

Re: BARX1 story: Accepted in Principle

From : "张力凡" <lfzya1@163.com>

To : "kailongli" <kailongli@pku.edu.cn>

Agree

6. Guoshun An

Re: BARX1 story: Accepted in Principle

From : "安国顺" <guoshunan@bjmu.edu.cn>

To : "kailongli" <kailongli@pku.edu.cn>

Cc : 673048571@qq.com lizhenqiu0808@163.com

Agree

7. Juhua Ni

Re:BARX1 story: Accepted in Principle

From : "Ni Juhua" <juhuani@bjmu.edu.cn>

To : "kailongli" <kailongli@pku.edu.cn>

Cc : "673048571" <673048571@qq.com> "lizhenqiu0808" <lizhenqiu0808@163.com>

Agree

8. Hongti Jia

Re:BARX1 story: Accepted in Principle

From : "贾弘?" <jiahongti@bjmu.edu.cn>

To : "kailongli" <kailongli@pku.edu.cn>

agree

9. Shuyan Li

Re: BARX1 story: Accepted in Principle

From : "lishuyan" <shuyanli@bjmu.edu.cn>

To : "kailongli" <kailongli@pku.edu.cn>

Agree
