# Supplementary figures and images for: ZFP36 loss-mediated BARX1 stabilization promotes malignant phenotypes by transactivating master oncogenes in NSCLC
Source: Cell Death Dis. 2023 Aug 16;14(8):527. doi: 10.1038/s41419-023-06044-z (PMC10432398; doi:10.1038/s41419-023-06044-z)

Fig1-H

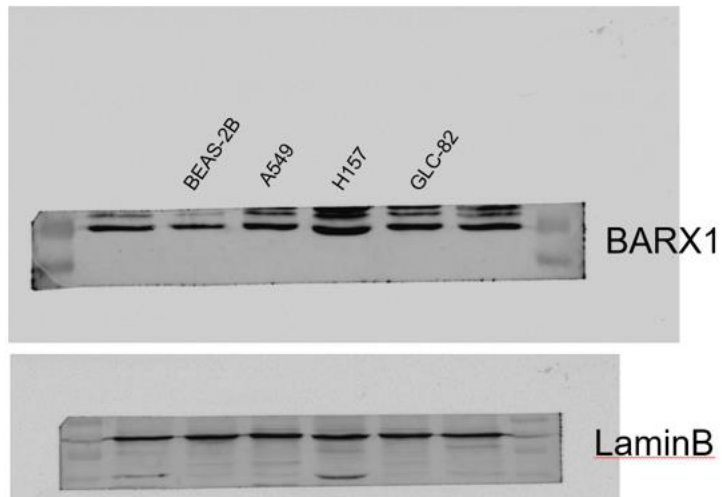

Figure4A-B

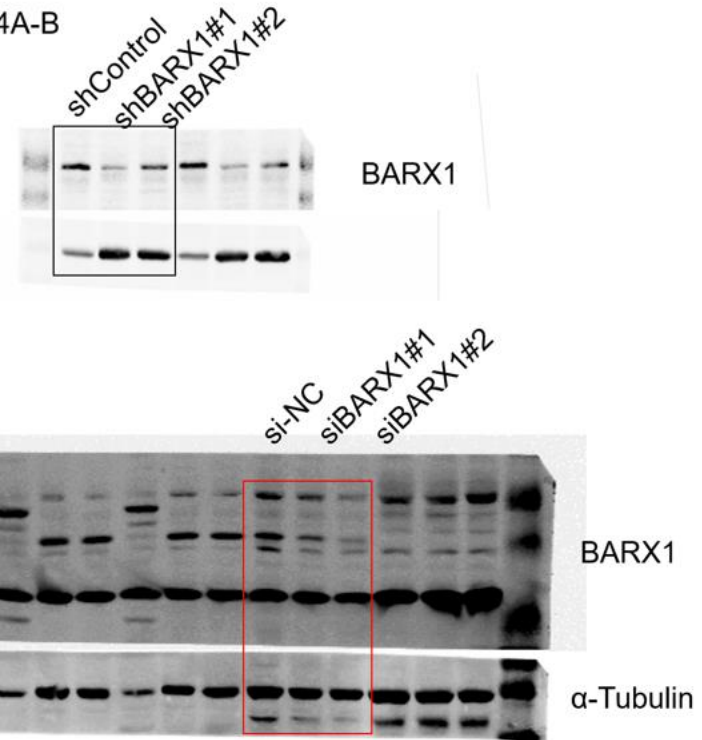

Figure3-A

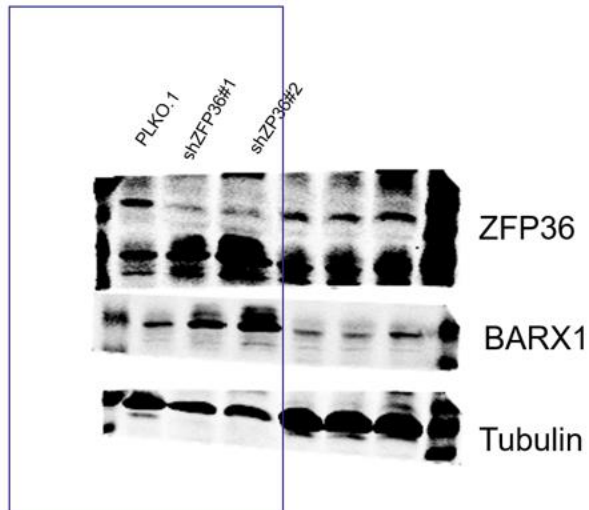

Figure3-C

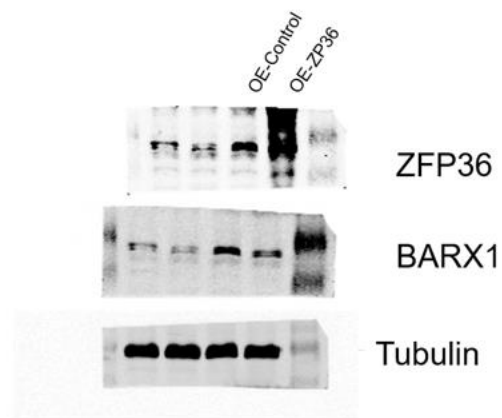

Supplement: Supplementary file 5 — Original Data File [file 41419_2023_6044_MOESM5_ESM.pdf]
